# “They Destroy the Reproductive System”: Exploring the Belief that Modern Contraceptive Use Causes Infertility

**DOI:** 10.1111/sifp.12076

**Published:** 2018-11-09

**Authors:** Erica Sedlander, Jeffrey B. Bingenheimer, Mary Thiongo, Peter Gichangi, Rajiv N. Rimal, Mark Edberg, Wolfgang Munar

## Abstract

A common reason for nonuse of modern contraceptives is concern about side effects and health complications. This article provides a detailed characterization of the belief that modern contraceptives cause infertility, and an examination of how this belief arises and spreads, and why it is so salient. We conducted focus group discussions and key informant interviews in three rural communities along Kenya's eastern coast, and identified the following themes: (1) the belief that using modern contraception at a young age or before childbirth can make women infertile is widespread; (2) according to this belief, the most commonly used methods in the community were linked to infertility; (3) when women observe other women who cannot get pregnant after using modern contraceptives, they attribute the infertility to the use of contraception; (4) within the communities, the primary goal of marriage is childbirth and thus community approval is rigidly tied to childbearing; and, therefore (5) the social consequences of infertility are devastating. These findings may help inform the design of programs to address this belief and reduce unmet need.

Reducing unmet need for modern contraceptives remains a top priority for organizations working in global health and international development. Couples can use modern contraceptives to delay the onset of childbearing, to space births, and to limit completed family size. This in turn can lead to benefits at the individual, family, community, and national levels, including reduced maternal, infant, and child mortality; the reduction of poverty; increased education for girls and boys; improvements in the status of women; and economic growth (Canning and Schultz 2012; Cleland et al. 2012; Conde‐Agudelo et al. 2012; Bongaarts 2016; Miller and Babiarz 2016).

Nevertheless, the actual level of modern contraceptive use remains well below its potential. This is especially true in sub‐Saharan Africa, where the modern contraceptive prevalence rate lags behind other regions of the world and unmet need for modern contraceptives remains high (Sedgh and Hussain 2014). Accordingly, the UK Department for International Development, the Bill and Melinda Gates Foundation, and other organizations in 2012 adopted the goal of increasing the number of women using contraception by 120 million by the year 2020 (Brown et al. 2014). The United States Agency for International Development (USAID) set a similarly ambitious target (Fabic et al. 2015).

The key question is how to accomplish these goals. While numerous studies over decades have documented factors that contribute to unmet need, a recent study summarized the reasons using Demographic and Health Survey (DHS) data from 51 countries between 2006 and 2013 (Sedgh and Hussain 2014). Among the African countries included in the study, the most common reason for nonuse was concern about side effects or health complications. Such concerns accounted for 28 percent of nonuse in Africa, and a staggering 43 percent of nonuse in Kenya, where our study is based. An important limitation of that study (and of the DHS methodology on this topic) is that the category “side effects and health concerns” may encompass a wide range of factors, including scientifically established side effects of specific methods as well as various myths or misconceptions.

Qualitative studies in Kenya and elsewhere in the sub‐Saharan Africa region provide a modest amount of additional detail on what the category “side effects and health concerns” may include. One such factor is the belief that modern contraceptive use causes infertility. This is important to understand given that motherhood often defines a woman's treatment in the community and infertility is seen as primarily a woman's issue (Kimani and Olenja 2001; Hollos et al. 2009). Therefore, the destructive social consequences of infertility in sub‐Saharan Africa for women are immense (Donkor and Sandall 2007; Naab, Brown, and Heidrich 2013; Rouchou 2013). In qualitative studies in Ghana (Adongo et al. 2014; Hindin, McGough, and Adanu 2014), Kenya (Burke and Ambasa‐Shisanya 2011; Ochako et al. 2015), Madagascar (Klinger and Asgary 2017), Nigeria (Otiode, Oronsaye, and Okonofua 2001; Schwandt et al. 2015), Rwanda (Farmer et al. 2015), and Uganda (Morse et al. 2014), participants mentioned infertility along with other side effects as reasons for nonuse. In some cases, infertility is mentioned only briefly. Some studies, however, provide slightly more detail about the nature of this belief. In western Kenya, for example, Burke and Ambasa‐Shisanya (2011) documented the belief that infertility can occur as a result of extended (five years or longer) use of injectables. Others (Hindin, McGough, and Adanu 2014; Ochako et al. 2015) documented the belief that use of hormonal methods by young, nulliparous women is most likely to lead to infertility.

While prior qualitative studies have provided some detail on the nature of these beliefs, quantitative surveys provide some information about their prevalence. In a nationally representative sample in Nigeria, Ankomah, Anyanti, and Oladosu (2011) found that nearly one‐third agreed with the statement, “Family planning can lead to infertility in a woman.” Surveys in Kenya, Nigeria, and Senegal demonstrated that the percentage of women endorsing the belief that “Contraceptives can harm your womb” ranged from 62 percent in Kenya to 37 percent in Senegal (Gueye et al. 2015). Similar beliefs were also documented in a survey in northern Ethiopia (Gebremariam and Addissie 2014).

Addressing the belief that modern contraceptive use causes infertility may be an important component to increase uptake of contraception and to reduce unmet need. To be truly effective, however, these interventions must be informed by a deeper understanding of this belief. Providing this deeper examination of the belief that contraception causes infertility is the goal of this article. Drawing upon data from focus group discussions (FGDs) and key informant interviews (KIIs) conducted as part of a larger mixed‐methods study on social networks, social norms, and uptake of modern contraceptives in rural Kilifi County, Kenya we describe in greater detail prevailing beliefs about the link between modern contraceptive use and infertility. By connecting this belief to other themes in the data, we also provide some insight into how these beliefs may arise and spread, and into the factors that may make them salient to people in this and other sub‐Saharan African settings.

## THEORETICAL FRAMEWORK

The broader project from which this article derives was shaped by the Diffusion of Innovations theory (Rogers 2003) and by efforts to integrate the science of social networks with the science of social influence (Contractor and DeChurch 2014). The core idea is that modern contraceptive use cannot spread from person to person in the same way that a pathogen like HIV does. Rather, what may spread directly from person to person and diffuse through social networks are beliefs, attitudes, and social norms that influence the likelihood of using or not using modern contraceptives (Casterline 2001). The overall goals of the project include the following: identify the beliefs, attitudes, and social norms that spread in these communities; delineate the social process by which the transmission occurs; describe the structure of the social network where these ideas spread; and model the spread of the ideas and of modern contraceptive use itself. Two constructs from *Diffusion of Innovations* (Rogers 2003) were particularly important in our conceptual approach. The first, *compatibility*, posits that innovations will spread through populations to the extent that they are consistent with the values, beliefs, and needs of intended users. This construct relates especially to fertility desires, which historically have been very high in sub‐Saharan African societies but have been declining in recent decades (Casterline and Agyei‐Mensah 2017). The second, *relative advantage*, involves the extent to which the innovation is perceived to provide a superior means to achieving a given end than other alternatives. Compared to traditional methods of avoiding births such as withdrawal and periodic abstinence, modern methods such as injectables may offer advantages including higher effectiveness and greater potential for control by women, but may have disadvantages that include side effects, costs, and other factors (Rossier and Corker 2017). Thus, the beliefs, attitudes, and social norms whose spread we sought to understand included not only those relating directly to modern contraceptives, but also those pertaining to the desirability of delaying, spacing, or limiting births.

## METHODS

### Study Setting

Kilifi County includes both rural and semi‐urban populations, and the capital is 56 kilometers northeast by road from the city of Mombasa. The predominant local language is Giriama, but Kiswahili is also widely spoken. Kilifi has some of the highest poverty levels, lowest literacy rates, and highest indicators of gender inequity nationally (Molyneux, Peshu, and Marsh 2005; Molyneux et al. 2007). The total fertility rate is 5.1 children per woman, 99.7 percent of women have heard of contraception, and 34.1 percent of currently married women ages 15–49 use contraception (National Bureau of Statistics 2014).

### Data Collection Modalities

We chose qualitative research methods for this study because we wanted to understand why women in this community are or are not using family planning. Qualitative inquiry can improve the description and explanation of complex, real‐world phenomena related to attitude and behavior change (Greenhalgh 2016). For this study, we collected two types of qualitative data, KIIs and FGDs, as complementary strategies. We conducted semi‐structured interviews with key informants because they provided a critical vantage point into the community as a whole. Key informants are, by definition, individuals who are likely to have broad as well as in‐depth knowledge at the community level. KIIs also provided the deep dive necessary to answer our research question rather than breadth that can be achieved from focus groups. We also conducted FGDs with men, women, and adolescent boys and girls from the community to obtain a snapshot of everyday practices, beliefs, and knowledge. We stratified FGDs by village, gender, and age. We chose a homogeneous sampling method to reduce variation, to facilitate group discussion, and to describe a particular subgroup in depth (Patton 2002).

### Instrument Development

We developed the interview and focus group guides (Appendix A) based on the project's theoretical framework (Rogers 2003; Contractor and DeChurch 2014), a review of the literature, detailed feedback from co‐investigators in Mombasa and Nairobi, Kenya and our previous work on contraception uptake in Ethiopia (Sedlander et al. 2018). Additionally, we conducted five pilot interviews with key informants and three focus groups from nearby villages to ensure questions captured what we intended and were culturally relevant. Three pilot interviews were transcribed for further analysis and then guides were modified to improve clarity. Interview guides covered barriers to and facilitators of family planning, and attitudes and beliefs about childbearing and fertility. To explore family planning norms in a less personal and sensitive way within the focus groups, we used vignettes, short stories about hypothetical characters that live in a rural village in Kilifi County (Gourlay et al. 2014).

### Sampling

We used a random sampling procedure to select participants for FGDs. Researchers conducted a household enumeration activity in each of the three villages. Based on the number of individuals needed for FGDs, against a sampling frame that consisted of the entire village, we used a proportional skip pattern that began with a randomly selected initial participant to identify households from which to select every succeeding participant for each category (e.g., mothers, adolescent boys). We used purposive, critical case sampling to select key informants who included village chiefs and elders, pastors, teachers, and health care workers, based on their level of knowledge about family planning use in the village (Patton 1999). Local village chiefs helped us sample the most knowledgeable key informants.

### Data Collection

In May and June of 2017, we conducted a total of 21 FGDs and 10 KIIs (N=163). See Appendix B for number of total interviews and focus groups by subpopulation. Our study team conducted a two‐week training in Mombasa, Kenya that included mock interviews and ethics on human subject's research. We recruited research assistants based on previous experience and training in conducting qualitative interviews. The trained Research Assistants (many of whom live in Kilifi County) conducted all of the interviews. For each interview, we matched the gender of interviewer and interviewee. We selected three rural communities for our study, one with a relatively high modern contraceptive prevalence rate (MCPR) of 44 percent according to data from a 2015 Performance Monitoring and Accountability (PMA2020) survey; one with a very low MCPR of 10 percent; and one with an intermediate MCPR of 29 percent, which is close to that of the county as a whole (33 percent) (PMA2020 2015). For each FGD, one member of the research staff observed the group and took field notes, and researchers wrote field notes following each KII. All interviews were conducted face‐to‐face in Kiswahili, by native speakers trained in qualitative interviewing. Audio recordings were then transcribed in Kiswahili and translated into English for analysis. Participants were also asked to complete a form collecting demographic information (see Table 1).

**Table 1:** Description of the sample including men, women, adolescent boys and girls, Kilifi County, Kenya

	(N = 163)	
	Mean (Range)	Percent
Age	26.2 (13–65)	
Female		65.2
Education		
None		14.1
Incomplete primary		47.2
Complete primary		26.9
Incomplete secondary		3.6
Complete secondary or higher		7.9
Marital status		
Currently married		61.9
Not married		34.9
Living with a partner		2.5
Religion		
Christian		52.7
Muslim		43.5
Other		3.6
Children		
None		40.4
One, two, or three		29.4
Four or more		29.4
Contraception use in village among married women		
High		42.9
Medium		13.4
Low		44.1

### Analysis

Two researchers, ES and JB, conducted thematic analysis to characterize the attitudes and beliefs relevant to fertility and family planning. Prior to beginning analysis, ES and JB wrote reflexivity memos to acknowledge their experiences and opinions in an effort to reduce individual biases. The process of writing and sharing these memos created a greater awareness of how our background may affect the analysis (Miles and Huberman 2014). We used an iterative approach to data collection and analysis, whereby analysis and data collection took place concurrently. This allowed us to determine when saturation occurred and no new themes were emerging from the data. We independently reviewed transcripts to identify initial themes and to develop a code book. We used both inductive and deductive coding. Specific *a priori* codes were used to identify text related to attitudes, beliefs, and practices associated with fertility and family planning, and additional codes were added to the code book based on new themes that emerged during coding. We identified themes based on the following criteria: (1) frequent recurrence within a single interview, (2) prevalence across interviews, or (3) intensity with which participants discussed this theme (Bazeley and Jackson 2013). We uploaded transcripts and the code book into NVivo v.11 for analysis. One researcher, ES, coded all transcripts and another, JB, coded 20 percent to ensure consistency across coders. These researchers met regularly over the course of analysis to discuss codes, review memos, reconcile discrepancies, and compare emerging themes. The researchers most closely involved in data analysis also met with the wider research team (ME, MT, PG, RR, and WM), to discuss emerging themes. Using NVivo, we compared codes and content across sources, ran specific word queries to find associations between themes, and created hierarchal visual displays of codes to identify linkages and patterns in the data. This study was approved by Institutional Review Boards at the George Washington University and the University of Nairobi.

## RESULTS

Several themes in the data describe why and how the belief that contraception causes infertility is so salient in the study settings: (1) the belief that using contraception at a young age or before childbirth can make women infertile is widespread; (2) according to this belief, the most commonly used methods in the community were linked to infertility; (3) women observe other women who cannot get pregnant after using contraception and attribute the apparent infertility to modern contraceptive use; (4) the primary goal of marriage is childbirth and community approval is rigidly tied to childbearing; and, therefore (5) the social consequences of infertility are devastating.

Of all the side effects that participants mentioned, the belief that modern contraceptive use can *cause* infertility by *“spoiling your reproductive system”* was the most frequently mentioned (84 percent of KIIs/FGDs referenced side effects, and from those all of them mentioned infertility). Participants also referenced specific beliefs about *how* contraception can make women infertile. A common belief was that using it at a young age or before bearing at least one child can make *“your womb weak.”* Many other participants mentioned that not only will using it before marriage *“block your uterus”* but also everyone will know *“what she had being doing.”* In other words, her *“barren‐ness”* will be proof that she used contraception and had sexual intercourse before marriage.

Additionally, we asked about use of contraception for adolescents and many participants stated that, *“she will have reproductive health complications because her uterus is not yet developed.”* Another participant reiterated this sentiment, *“The teenagers who use that needle for a long time, and then they get married, when they try to get pregnant, the pregnancy pours (a reference to miscarriage), or they don't get pregnant at all.”* Lastly, participants expanded on this belief stating that if a woman uses family planning before she has her first child, she will not know whether she is *“fertile”* or *“able to know themselves.”* Therefore, waiting until after having one or two children is the preferred time to begin to use contraception. This will allow a woman *“to assess your fertility.”* One participant reiterated this sentiment, *“After you have given birth, you can plan.”* Clearly, participants were more open to using contraception to space or limit after they had proven their fertility.

Additionally, we examined whether or not certain methods of contraception were perceived to be more associated with causing infertility than others. Participants discussed both oral contraceptives and injections as the only methods specifically linked to infertility, but those methods are also the most widely used in the communities. A female from a focus group said, *“When injected you cannot bear children, you become barren, you will never be able to give birth again.”* The same belief was discussed with oral contraceptive pills, *“They say if you use pills, when you want a child it becomes a problem.”* Multiple men from focus groups reported the same, “You mean those tablets. They say they cause damage to the uterus.” On the other hand, only a couple of participants mentioned that the IUD is the only method that does *not* cause infertility. One participant said, *“The coil, the Copper T, that is the one that is said not to have harm, but all these others, all of them are harmful. When you want a child, it will disturb you.”* Ultimately, there was not a clear preference for one method over another despite two references to the IUD.

Furthermore, we examined how beliefs about the connection between contraception and infertility fall on a spectrum from personally believing to personally disavowing this belief. While most participants mentioned that contraception causes infertility, not everyone reported that they personally believe that it does so, but simply that this belief exists within the community. Of those who mentioned the belief that contraception causes infertility, approximately 60 percent affirmed the belief. For example, a young man from a focus group said, *“She might use family planning and when they are removed from her body, she is not able to conceive. Because those drugs affect the inside and the uterus becomes thin such that it can't carry a pregnancy.”* Twenty percent of respondents simply described the belief that others held. A female from a focus group reported that, *“Some people do say they destroy the reproductive system.”* Lastly, approximately 13 percent of study participants completely renounced this belief. A male pastor said, *“There is that perception that those who use family planning before getting married, it will affect them later, though it's a perception and has no witness.”* Overall, we found that more key informants denounced the belief than focus group participants. This is not surprising given that key informants tend to be more educated.

Additionally, we examined how beliefs that contraception causes infertility emerge. We discovered that men and women reported observing someone within the community who used contraception and then could not give birth. It is important to note that, to reduce social desirability bias, many of the questions were vignette‐based, and so participants were talking in the third person about what might happen to this fictional character in the community. *“Another reason* [that she will not use contraception] *is that she will see her peer has used those drugs* [pills] *and she is now trying to conceive and she cannot.”* A health care provider from a low contraception village reiterated this, *“Now you see another is afraid to come* [to the health center to get contraception] *as a result of that alarm* [around infertility] *that was raised in the community.”* It is important to note that we did not ask specifically about how long couples could not conceive so we do not know if perceptions that contraception can make you infertile are in fact a case of infertility or simply within the normal range of time that it takes a couple to conceive.

While the topic of infertility mainly arose when we asked KIIs and FGD participants about the advantages and disadvantages of family planning, it also arose several times in another context—when participants were asked about whether a newly married couple should conceive immediately or allow some time to pass before becoming pregnant. The vast majority of participants indicated that couples should conceive very soon after marriage, and many stated that failure to do so could raise concerns about infertility. As one FGD participant put it, *“Aah having children is an important thing because you will get to know that you are complete, you are not barren because you have children.”* Conversely, participants indicated that the absence of a pregnancy soon after marriage often leads to “talking” and “questions.” As one key informant stated, “*Because if you stay for some months and your wife has not gotten pregnant, they will start asking, ‘how is that, how is that?’”*

Infertility was not, however, the only plausible explanation provided for not becoming pregnant soon after marrying. One explanation is using contraception to plan your family. In the absence of an acceptable explanation like this, however, respondents believed that people would begin to worry about infertility. As one key informant described, family members may intervene: *“So if you go past that time, you will be disturbed, you will be told, ‘Wait, we will bring you certain medications.’ This will be brought and boiled. Some drums will be beaten and you will dance to them.”*

These concerns about infertility can become particularly intense because, beyond simply wanting to have children, many people see childbearing as the fundamental purpose of marriage. As one FGD participant put it, *“The reason they got married is so that they would have children”* and *“When you are married it is required that you get a child.”* Having children is a matter that involves not just the couple themselves, but also the entire family, especially on the husband's side. As one FGD participant said, *“After getting married, your parents will be expecting and start counting if it is a month or two months, so that they can start seeing some signs of a child from you.”* A woman who marries and then does not bear a child may be seen as failing to uphold her side of the marital bargain. According to FGD participants, people would sometimes describe such a wife as someone who was *“just filling the toilet here”* or who *“just came here to eat for free.”* In addition to these negative judgments, failure to produce a child could also lead to the end of a marriage and the wife being “sent back to her family” because “*there is no need to live with an infertile woman.”* Another said, *“He can be told to leave her and marry another one.”*

The view that childbearing is the fundamental purpose of marriage and the most important obligation that a wife has to her husband and family, appeared to be widespread among FGD participants but it was not completely ubiquitous. As one key informant, a pastor, put it, *“The goal of marriage is not only to get children. There are other responsibilities that you need to perform.”* The same theme was echoed by an FGD participant who explained that a couple may use contraception because,*“Maybe they did not marry with the aim of having children.”* Relatedly, some participants articulated views of marriage that were more emotionally motivated than is traditional. One KII participant, asked about a couple using family planning to delay pregnancy after marrying, said, *“The benefit they get is knowing each other for that time that they still don't have children.”* And an FGD participant described how such a couple might deal with outside pressure to begin childbearing immediately: *“They should persevere each other because there are neighbors who will say, ‘You have stayed for two or three years and you have not gotten a child’… so you must have planned with your husband and agreed.”* Such views, however, were in the minority.

## DISCUSSION

In this study, we found that the belief that contraceptives cause infertility is both widespread and salient in rural Kilifi County, Kenya. The main belief is that contraception, especially when used at a young age and/or prior to bearing children, can weaken a woman's uterus or otherwise damage her reproductive system, making it difficult or impossible for her to conceive or to carry a pregnancy to term in the future. The two methods that are most widely used in the study communities—oral contraceptive pills and injectables—were the only methods implicated in the belief. Some participants articulated this as their own belief, but many described it as a belief that is prevalent in the community without explicitly taking a position on its credibility; only a few explicitly disavowed the belief. We also found that fertility and infertility are key preoccupations of the families of newly married couples, as many people see childbearing as the fundamental purpose of marriage and the most important responsibility of a wife to her marital family.

This is far from the first study to document the importance of fertility and the negative social implications of infertility for women in sub‐Saharan African societies. Summarizing decades of research from around the region, Caldwell and Caldwell (1987: p. 418) wrote, “The horror of barrenness is associated with the experience of the barren as well as with the underlying fear rising from its interpretation.” More recent research in Kenya (Kimani and Olenja 2001) and elsewhere in sub‐Saharan Africa (Hollos and Larsen 2008; Hollos et al. 2009) suggest that primary infertility—that is, the infertility of a woman who has not yet borne any children—remains a source of economic and social devastation for those who experience it, with such women being seen as unacceptable for marriage and prevented from attaining the status of full womanhood. Nor is primary infertility extremely rare. A recent review (Mascarenhas et al. 2012) put its prevalence at around 2 percent among child‐seeking women in sub‐Saharan Africa. This may help to explain both the prevalence and the salience of the belief that contraceptive use among nulliparous women can lead to infertility. Instances of primary infertility are not entirely rare, and when they occur they may draw the attention not only of the couple and their family but of the community as a whole. On the other hand, simply taking the normal time to conceive may be confused with infertility. Our study did not examine whether perceptions about infertility were real or not.

Additionally, the severe social consequences of infertility may help explain the salience of these concerns to people in rural Kilifi County. Most theoretical approaches to risk perceptions (Slovic 1987; Champion and Skinner 2008) classify those perceptions into two components: the perceived influence of a specified behavior on the likelihood of a negative outcome, and the severity of that outcome. Even highly unlikely outcomes can cause substantial concern and influence behaviors if those outcomes are perceived to be sufficiently severe. Our data suggest that respondents do not believe that every nulliparous woman who uses modern contraceptives will become infertile. The belief, instead, appears to be that modern contraceptive use increases one's risk of infertility by an unspecified amount. Because the social consequences of primary infertility are so severe, however, even a small and uncertain incremental perceived increase in the risk of infertility may be sufficient to motivate people to avoid using modern contraceptives.

Moreover, fully understanding the salience of concerns about infertility to people's decisions to use or not use modern contraceptives may require us to go beyond expectations about concrete health and social consequences. Given that motherhood is central to a woman's identity in many sub‐Saharan African settings, Kahan's (2007) Cultural Identity Framework may provide deeper insight. This framework states, in essence, that individuals’ perceptions of the risks associated with specific behaviors or exposures is shaped not only by information they receive about those risks, but also by the meaning that those behaviors or exposures have in relation to a culturally defined identity. This framework can elucidate how and why individuals’ risk perceptions concerning a particular behavior differ (e.g., contraceptive use). The framework examines how cultural views affect risk perception in patterns that suggest cultural‐identity protection. For example, in a community where motherhood is highly prized and infertility threatens this role, the risk of infertility may be perceived as much higher than it actually is and the fear of unintended pregnancy may be perceived as much lower. In this sense, the salience of the belief that modern contraceptives can cause infertility may transcend concrete outcome expectations and reflect a deeper discomfort with the concept of deliberately attempting to prevent a pregnancy, which is linked to widely held norms and values about the centrality of motherhood to womanhood within African (and other) social systems.

Our data also provide some indication that the belief about a link between contraceptive use and infertility spread through informal social networks. This is consistent with other findings about the roles of gossip and informal conversations in the spread of information (and misinformation) about family planning in Kenya (Rutenberg and Watkins 1997) and elsewhere in sub‐Saharan Africa (Paz Soldan 2004). That model would appear to offer a convincing explanation for how ideas about perceived side effects of modern contraceptive use spread within communities. In contrast, beliefs about a link between contraceptive use and infertility, similar to those articulated by our study participants in rural Kilifi County, have also been documented elsewhere in Kenya (Burke and Ambasa‐Shisanya 2011; Ochako et al. 2015) as well as in other East African countries (Farmer et al. 2015), and Uganda (Morse et al. 2014), thousands of miles away in West Africa (Otiode, Oronsaye, and Okonofua 2001; Richards 2002; Adongo et al. 2014; Hindin, McGough, and Adanu 2014; Schwandt et al. 2015), and even in the island nation of Madagascar (Klinger and Asgary 2017). It is not obvious how such a uniform set of beliefs could spread so widely without being amplified in some way, such as by mass media. While many people mentioned that they hear about family planning via television and radio advertisements, we found no evidence that people acquired their beliefs about a link between modern contraceptive use and infertility via mass media. This suggests that the belief may arise independently and spread locally in settings throughout the region.

## LIMITATIONS

Our study has some limitations that may impinge upon the interpretation of the results. One is that our study is limited to three rural communities in Kilifi County, Kenya so it may not be representative of sub‐Saharan Africa as a whole, Kenya, or even urban areas of Kilifi County. Our purpose, however, focused more on documenting the barriers than establishing their representativeness. Second, while we reported differences in attitudes among key informants and FGD participants, we did not report differences between age and gender. A quantitative study design may be better suited to make this comparison. Additionally, we did not examine whether or not reported infertility in the community was real or simply perceived, as our qualitative study design is not ideal to examine real infertility prevalence within the community. Furthermore, our KII and FDG guides did not focus exclusively on beliefs about the link between modern contraception and infertility. Rather, these topics arose in response to more general questions about family planning and reproduction and a deeper exploration of the beliefs and attitudes underlying the various reasons for nonuse. One advantage of this is that it provides some indication of the salience of these beliefs: we did not directly ask participants about them but they were still prominent. On the other hand, a narrower focus on these beliefs could have enabled us to characterize them in greater detail, and to gain more insight into the processes by which they arise and spread in the community. Perhaps this should be the focus of further study. Given that other reasons for nonuse have been consistently identified in various studies (e.g., opposition from a significant other, infrequent or no sex, etc.), a comprehensive characterization of nonuse would require a better understanding of the interdependencies and feedback effects that can plausibly exist among these various reasons for nonuse. Additionally, infertility is not the only perceived negative side effect that nonusers mention in various surveys. Some of the other side effects related to contraception use in general discussed in this study include growing “meat,” “developing a growth” (references to cancer and fibroids), “an abnormal child,” and “constant monthly period bleeding.”

## IMPLICATIONS

In spite of these limitations, our findings have implications for strategies to increase the uptake of contraceptives in Kilifi County and perhaps more generally in Kenya and even sub‐Saharan Africa as a whole. In general, it may be useful for interventions to address the belief that modern contraceptive use causes infertility. Some potential mediums to consider are one‐on‐one counseling with patients in family planning clinics, community‐based group education programs, or mass media campaigns. While communication campaigns have addressed side effects broadly, to our knowledge past communication programs have rarely if ever directly addressed the belief that contraceptive use causes infertility (Halperin et al. 2013; Burke and Ambasa‐Shisanya 2014; Krenn et al. 2014). The message itself could acknowledge that using contraceptives and becoming a mother are not mutually exclusive, perhaps by role modeling mothers who have previously used family planning methods. However, crafting messages that debunk prevalent beliefs is difficult. Research in cognitive psychology and communication sciences suggests that efforts to correct misperceptions and debunk myths are often ineffective, and some even backfire (Lewandowski et al. 2012). This may be especially true when the myths help support highly valued cultural identities and worldviews (such as motherhood as the highest calling), when the issue has high ego‐involvement (Rhine and Severance 1970), and when the correct information may be interpreted as threatening (hampering the ability to conceive) (Kahan et al. 2007; Nyhan et al. 2014). Our findings that men and women view the risk of infertility as greater than the risk of unintended pregnancy align with these theories. Because infertility appears to have devastating social consequences (and unintended pregnancy does not), the perceived risk of anything that may affect fertility, in this case the use of contraception, is extremely high. In other words, the belief that reproduction is part of one's cultural identity (for both males and females) may moderate fear of infertility. Future studies may want to test this hypothesis to be able to tailor their message and segment their audience accordingly.

As others have previously suggested (e.g., Inhorn 1994; Richards 2002), programs intended to promote uptake of contraceptives in settings where fertility is highly valued but infertility is widespread may ultimately prove more effective if they also make a concerted effort to address infertility. A recent review (PATH 2015) provides several useful recommendations. For example, it may not be enough to simply debunk a myth without also replacing the myth with a compelling alternative explanation (Lewandowsky et al. 2012). Thus, rather than merely stating that modern contraceptives do not cause infertility, it may be useful for interventions to include information about the actual causes of infertility.

In closing, treating reproductive health choices as individual decisions, without taking into account the social and community pressures impinging on a woman, will have limited impact on a woman's reproductive autonomy (Horne, Dodoo, and Dodoo 2013). It is critical for policy‐ and behavior‐change program designers to understand and mitigate the impact that the fear of infertility has on a woman's desire to use contraception. Increasing efforts to address infertility would bring reproductive health programs in line with the voluntary, human‐rights based approach to family planning (Hardee et al. 2014), and this approach may also be less threatening to cultural values and thus earn greater levels of community buy‐in. If interventions succeed in reducing the prevalence of the belief that contraceptives cause infertility in nulliparous young women, this could increase use of contraceptives, moving the world closer to FP2020 and other goals (Brown et al. 2014; Fabic et al. 2015).

## Appendix A

### Focus Group Guide

#### Before beginning the interview

Welcome participant and introduce yourself.

Hello, and thank you for coming today. My name is _____________________ and I am a (your role) at ICRHK. I am conducting a research study to understand the attitudes and beliefs that affect family planning use in Kilifi County.

Thank you so much for agreeing to participate and taking time out of your day.

Review key points, ethics, and confidentiality policy:

- You will not gain any direct benefit from participating in this research, however we hope our results will be used to improve public health programs for women.
- We do not anticipate you will experience any risks but please feel free to not answer any questions that you are uncomfortable with or to stop the interview at any time.
- The discussion in this interview is completely confidential.
- Your responses will be kept confidential and your name will not be cited on any written materials coming out of this study.
- The interview is being recorded so that we can accurately capture what you are saying, but no one besides the research team will have access to it.
- There are no right or wrong answers to the questions I am going to ask you. All experiences are important and valid.
- We expect the interview to last about one and a half hours.

Do you have any questions about the research and your participation before we start?

#### Warm‐up Questions

I'm new to this community (name village). Can you tell me a little bit about it? Like what do people do to earn money? Is this a big village or a small one?

#### Importance of Having Children in General

So today we're going to talk about women and children. What are the good things about having children? What are the difficult things about having children?

#### Delay and Initiation of Childbearing

OK. Now we're going to make up a character and discuss her story. She is a female, 22 years of age, and she just got married to a neighbor who is a farmer. What should we call her and her husband? (Ask for suggestions and decide on a name together.)

- What are the ideal circumstances for (woman's name and man's name) to have their first child? (Probe: After a certain number of months or years? Anytime after marriage? At a specific age? When able to provide?)
- What are the benefits of waiting some time after they are married to have their first child? What are the difficulties that (woman's name) or the couple would face if they waited to have their first child? (Probe: What are the benefits of having a child soon after marriage?)
- Who influences (woman's name) decision to have a child? Who has the most influence about when (woman's name) should have a child? Why? (Probe: Her husband, family, etc.)
- Where might they get information or advice about when to have their first child?

#### Birth Spacing

OK. Now we're going to make up a new couple, a husband and wife, and discuss their story. What should we name them? OK. (Name given) and her husband (name given) have one child, a girl, who just turned one year. After the first child, some families have a second one right away and others wait for a while. Can you tell me why (names given) might wait or not?

Why might (names given) want to wait for some time between having one child and having the next? Why might they want to have their next child as soon as possible?

Apart from (names given) themselves, who might influence their decision about when to have another child? (Probe: Other people in the community, family or friends?)

Where might they get information or advice about when to have their next child? (Probe: Other people in the community, family or friends?)

#### Completed Family Size

How many children would a couple like this (names given) want to have? Why do you think they would want to have this many? Where might they get information or advice about when to stop having children?) (Reminder, still on vignette number 2, the couple who has one child who is a year old.)

Over the years, has anything changed about the number of children that women want to have? If yes, what are some of the reasons why the number of children that women want to have has changed? (Probe: Who may have influenced the change?)

What do you think (names given) believe are the advantages and disadvantages of limiting the number of children a woman has? (Probe: Who might influence their opinion? Probe: Other people in the community, family or friends?)

#### Family Planning/Modern Contraceptives: General Information

Which methods to prevent pregnancy would this couple consider? (Probe: Any others?)

Where would they first learn about family planning/contraception? (Probe: Other people in the community, family or friends?) How has this changed over time?

From what other sources might they hear about family planning? (Probe: Other people in the community, family or friends?), media (radio, TV, newspapers, etc.).

Who else might they discuss family planning with? (Probe: Other people in the community, family or friends?)

#### General Benefits of and Barriers to Using Contraception

What are some reasons they might want to use family planning? (Probe: To space births, limit family size, delay first birth, enjoy living with your partner, etc.)

How would they learn about these reasons for using family planning? (Probe: Other people in the community, family or friends?)

What are some reasons they might have for not wanting to use family planning? (Probe: Side effects, partner opposition, fear of infertility, wants more children, etc.)

How would they learn about these reasons? (Probe: Where, when, from whom?)

#### General Family Planning Use in the Community

OK. Now I'm going to ask you about other people in the community.

We're going to talk about benefits and drawbacks of adolescents using family planning. What are the benefits of adolescents using family planning? What are the drawbacks of adolescents using family planning? How familiar are adolescents with family planning? How do they learn about it? (Probe: If they ask what do you mean by adolescents, you can answer, by adolescents I mean boys and girls 13–17 years old. If they don't ask, you can leave it as adolescents.)

We're going to talk about benefits and drawbacks of young unmarried women using family planning. What are the benefits of young unmarried women using family planning? What are the drawbacks of young unmarried women using family planning? How do they learn about it? (Probe: By young unmarried women, I mean women who don't have children and are between the ages of 18 and 25 years.)

#### Closing

That is the end of the questions I have for you, but do you have anything else you'd like to add to the discussion?

As a reminder, please do not share anything we spoke about today with anyone outside of this group.

Any questions?

Thank you for your time.

### Individual Interview Guide for Key Informants

#### Before beginning the interview

Welcome participant and introduce yourself.

Hello, and thank you for coming today. My name is _____________________ and I am a (your role) at ICRHK. I am conducting a research study to understand the attitudes and beliefs that affect family planning use in Kilifi County. I'm interviewing some key informants in the field like yourself to understand how the community is using, and what it feels about, family planning.

Thank you so much for agreeing to participate and taking time out of your day.

Review key points, ethics, and confidentiality policy:

- You will not gain any direct benefit from participating in this research, however we hope our results will be used to improve public health programs for women.
- We do not anticipate you will experience any risks but please feel free to not answer any questions that you are uncomfortable with or to stop the interview at any time.
- The discussion in this interview is completely confidential.
- Your responses will be kept confidential and your name will not be cited on any written materials coming out of this study.
- The interview is being recorded so that we can accurately capture what you're saying, but no one besides the research team will have access to it.
- There are no right or wrong answers to the questions I am going to ask you. All experiences are important and valid.
- We expect the interview to last about 45 minutes.

Do you have any questions about the research and your participation before we start?

#### Warm‐up Questions

Can you tell me about your role? I'm new to this community (name village), can you tell me a little bit about it? Like what do people do to earn money? Is this a big village or a small one? What do people eat here?

#### Importance of Having Children in General

So today we're going to talk about women, children, and family planning. In this community, what are the good things about having children? What are the difficult things about having children?

#### Delay and Initiation of Childbearing

- In this community, what are the ideal circumstances for a couple to have their first child? (Probe: A certain number of months or years, anytime after marriage, a specific age, ability to provide?)
- What are the benefits of waiting some time after the couple is married to have their first child? What are the difficulties that a couple might face if they waited to have their first child? (Probe: What are the benefits of having a child soon after marriage?)
- Who influences a couple's decision to have a child? Who has the most influence about when a woman should have a child? Why? (Probe: Her family, friends, community, etc.)
- Where might a couple get information or advice about when to have their first child?

#### Birth Spacing

OK. What about a couple in this community that already has one child who just turned one year? After the first child, some families have a second one right away and others wait for a while. Can you tell me why a couple with one child might wait or not?

Why might they want to wait for some time between having one child and having the next? Why might they want to have their next child as soon as possible?

Apart from the couple themselves, who might influence their decision about when to have another child? (Probe: Other people in the community, family or friends?)

Where might they get information or advice about when to have their next child? (Probe: Other people in the community, family or friends?)

#### Completed Family Size

How many children do couples in this community want to have? Why do you think they want to have this number of children? Where might they get information or advice about when to stop having children?

Over the years, has anything changed about the number of children that women in the community want to have? If yes, what are some of the reasons why the number of children that women want to have has changed? (Probe: Who may have influenced the change?)

What do you think couples believe are the advantages and disadvantages of limiting the number of children a woman has? (Probe: Who might influence their opinion? Other people in the community, family or friends?)

#### Family Planning/Modern Contraceptives: General Information

Which methods to prevent pregnancy do couples in this community consider? (Probe: Any others?)

Where do couples first learn about family planning/contraception? (Probe: Other people in the community, family or friends?) How has this changed over time?

From what other sources might they hear about family planning? (Probe: Other people in the community, family or friends?), media (radio, TV, newspapers, etc.).

Who else might they discuss family planning with? (Probe: Other people in the community, family or friends?)

#### General Benefits of and Barriers to Using Contraception

What are some reasons that couples want to use family planning? (Probe: To space births, limit family size, delay first birth, enjoy living with partner, etc.)

How do couples in this community learn about these reasons for using family planning? (Probe: Other people in the community, family or friends?)

What are some reasons they might have for not wanting to use family planning? (Probe: Side effects, partner opposition, fear of infertility, wants more children, etc.)

How would they learn about these reasons? (Probe: Where, when, from whom?)

#### General Family Planning Use in the Community

OK, now I'm going to ask you about other people in the community.

We're going to talk about benefits and drawbacks of adolescents using family planning. What are the benefits of adolescents using family planning? What are the drawbacks of adolescents using family planning? How familiar are adolescents with family planning? How do they learn about it? (Probe: By adolescents, I mean boys and girls 13–17 years old.)

We're going to talk about benefits and drawbacks of young unmarried women using family planning. What are the benefits of young unmarried women using family planning? What are the drawbacks of young unmarried women using family planning? How do they learn about it? (Probe: By young unmarried women, I mean women who don't have children and are between the ages of 18 and 25 years old.)

What does the organization that you represent think about family planning? (Probe: Could be a health post, church or mosque, school, community, etc.)

#### Closing

That is the end of the questions I have for you, but do you have anything else you'd like to add to the discussion?

Any questions?

Thank you for your time.

## Appendix B

### Data collection modalities stratified by village, gender, and age

**Table:**

			Focus Groups					
Village cluster	Modern contraceptive use (MCPR)	Key Informant Interviews (KIIs)	Women aged 18–25 with 0–1 children	Women aged 22–45 with 2+ children	Unmarried adolescent girls aged 15–17	Unmarried adolescent boys aged 15–17	Fathers aged 18–34	Fathers aged 35–60
Low MCPR	Low: 10.3%	4	2	2	2	1	1	1
Medium MCPR	Medium: 28.8%	2	1	1	1	0	0	0
High MCPR	High: 44.4%	4	2	2	2	1	1	1
